# OCT1 Polyspecificity—Friend or Foe?

**DOI:** 10.3389/fphar.2021.698153

**Published:** 2021-06-02

**Authors:** Marleen J. Meyer, Mladen V. Tzvetkov

**Affiliations:** Institute of Pharmacology, Center of Drug Absorption and Transport (C_DAT), University Medicine Greifswald, Greifswald, Germany

**Keywords:** OCT1, SLC22A1, polyspecificity, structure-to-function relationship, ligand-transporter interaction, species differences, genetic variants

## Introduction

Polyspecificity is one of the most characteristic features of organic cation transporter OCT1 (SLC22A1). Already upon the initial cloning it was recognized that the OCT1-mediated uptake could be inhibited by a variety of substances with different chemical structures (Gründemann et al., 1994). Following the initially identified substrate TEA^+^, the organic cations MPP^+^ and ASP^+^ and endogenous compounds such as dopamine and histamine were described as substrates (Gründemann et al., 1994; Busch et al., 1996a; Busch et al., 1996b; Mehrens et al., 2000), showing that not only aliphatic, but also aromatic cations with variable structures could be OCT1 substrates. Currently, more than 150 organic cationic compounds with highly variable chemical structures, including also commonly used drugs like metformin, morphine, sumatriptan, fenoterol, and lamotrigine have been reported to be substrates of the organic cation transporter OCT1 (Wang et al., 2002; Dickens et al., 2012; Tzvetkov et al., 2013; Matthaei et al., 2016; Shen et al., 2016; Tzvetkov et al., 2018; Haberkorn et al., 2021).

However, polyspecific does not mean unspecific. We recently reported that small differences in the chemical structures of morphinan opioids lead to substantial differences in the inhibitory potency or even to the inability to interact with human OCT1 (Meyer et al., 2019).

Twenty-seven years after OCT1 discovery, the mechanisms conferring its polyspecificity are still poorly understood. Our current knowledge about the mechanisms of OCT1-mediated transport is predominantly based on mutagenesis experiments. Several amino acids have been suggested to be of key importance for substrate binding and/or translocation (Gorboulev et al., 1999; Gorboulev et al., 2005; Popp et al., 2005; Sturm et al., 2007; Volk et al., 2009). The most prominent thereof is Asp475 in transmembrane helix 11, which is generally accepted to play a key role by interacting with the positive charge of the substrate. However, OCT1 is thought to have multiple binding sites that may overlap between substrates (Gorboulev et al., 1999; Popp et al., 2005; Volk et al., 2009; Chen et al., 2017; Boxberger et al., 2018), but there is no crystal structure available, neither for OCT1, nor for any of the closely related proteins of the SLC22A family. The homology models used instead are based on distantly related transporters that share maximally 19.5% identity with human OCT1 (Koepsell, 2020). Therefore, the exact binding sites of the different OCT1 substrates remain unclear.

## OCT1 Polyspecificity as a Foe

In many regards, the polyspecificity of OCT1 represents a disadvantage. It complicates the experimental analysis and the interpretation of the obtained results.

Firstly, OCT1 polyspecificity is a hurdle when studying the mechanisms of transport. Most of our current knowledge about OCT1 structure-to-function relationships has been obtained using MPP^+^ or TEA^+^ as substrates (Gorboulev et al., 1999; Gorboulev et al., 2005; Popp et al., 2005; Volk et al., 2009; Keller et al., 2019). Already for these two substrates, substrate-specific differences in the role of key amino acids were reported. While mutation of Arg440Lys affected the affinity for TEA^+^ but not for MPP^+^, mutation of Phe160Ala affected the affinity for MPP^+^ but not for TEA^+^ (Gorboulev et al., 2018). This may not be surprising, considering the structural differences between the two substrates. On the contrary, different ligands can be expected to interact with different amino acids of the transporter. Thus, despite their name, the so-called model substrates may be of only limited use for predictions of substrate-transporter interactions for clinically relevant ligands. Therefore, at least until we better understand the structural mechanisms of OCT1 polyspecificity, structure-to-function relationships have to be established for each substrate separately. As a practical consequence, *in vitro* data on inhibitory potencies using OCT1 model substrates may be of only limited predictive value for drug-drug-interactions (DDIs) with the actual victim drug (Koepsell, 2015, 2020; Hermann, 2021).

Secondly, in addition to the difficulties of transferring structure-to-function findings between substrates, polyspecificity aggravates the transfer of findings between species. Most of the available structure-to-function data have been obtained studying rat Oct1 (Gorboulev et al., 1999; Gorboulev et al., 2005; Popp et al., 2005; Sturm et al., 2007; Volk et al., 2009). However, human and rat OCT1 differ in 120 amino acids and each of them may potentially cause differences in OCT1 function. There is not much data directly comparing rat and human OCT1, but the affinity and the capacity of metformin and thiamine transport have been shown to differ substantially between mouse and human OCT1 (Chen et al., 2014; Meyer et al., 2020). Based on these differences, up to 11-fold higher maximal metformin concentrations may be expected in mouse than in human liver and hepatic effects of metformin in humans may be overestimated (Meyer et al., 2020). This may explain why loss of OCT1 activity in humans does not affect metformin efficacy (Zhou et al., 2009; Dujic et al., 2017) in contrast to strong effects observed in OCT1 knock-out mice (Wang et al., 2002; Wang et al., 2003).

More importantly, due to OCT1 polyspecificity, the species differences are also substrate-specific. While OCT1 inhibition results in strong differences in the uptake of ondansetron and tropisetron between mouse and human hepatocytes, no differences were observed for sumatriptan and fenoterol (Morse et al., 2020). Therefore, next to the known differences in OCT1 organ expression between the species (Gründemann et al., 1994; Schweifer and Barlow, 1996; Gorboulev et al., 1997; Green et al., 1999), differences in transport activity have to be kept in mind when interpreting existing data from animal models and cannot not be generalized among the substrates.

Thirdly, polyspecificity leads to substrate-specific effects of genetic variants in OCT1. OCT1 is genetically highly variable and common genetic variants lead to a reduction or to a loss of OCT1 function (Kerb et al., 2002; Shu et al., 2003; Seitz et al., 2015). Some of these variants have substrate-specific effects. The most common OCT1 variant in Europeans and White Americans (Seitz et al., 2015), the deletion of Met420 (OCT1*2), reduces the uptake of metformin, morphine, tropisetron, and O-desmethyltramadol (O-DSMT) by more than 75% (Shu et al., 2007; Tzvetkov et al., 2011; Tzvetkov et al., 2012; Tzvetkov et al., 2013), but shows normal or only slightly reduced uptake of sumatriptan, cycloguanil, and debrisoquine (Saadatmand et al., 2012; Matthaei et al., 2016; Matthaei et al., 2019). Therefore, homozygous carriers of OCT1*2 have to be regarded as complete loss-of-function phenotypes (so-called poor OCT1 transporters) when tramadol is administrated and as fully active (extensive OCT1 transporters) when sumatriptan is administrated. Hence, individual OCT1 activity scores have to be substrate-specific and cannot be generalized. This complicates the use of OCT1 pharmacogenetics in the clinical routine and requires clinical studies for each substrate.

Finally, the polyspecificity of OCT1 questions the idea of one “ultimate” pharmacophore valid for OCT1 ligands. Indeed, the published ligand-based pharmacophore models of OCT1 differ in the number, type, and distance of their features. While the models of Bednarczyk et al. (2003), Moaddel *et al.* (Moaddel et al., 2005; Moaddel et al., 2007) and from our group (Meyer et al., 2019) show some resemblance, the model by Nies et al. (2011) shows more pronounced differences, the most striking being the absence of a positively ionizable site. This is not surprising and may simply reflect the coexistence of different binding sites in OCT1. Therefore, before we understand which ligands bind to which binding sites, it will be difficult to correctly identify chemical features necessary for interaction with OCT1.

## OCT1 Polyspecificity as a Friend

When utilized properly, the polyspecificity of OCT1 can also be an ally in understanding the transport mechanisms of OCT1. Many of the disadvantages listed above can be turned into experimental tools to study polyspecificity.

Firstly, the *in vitro* prediction of DDIs should be performed with more than one (victim) substrate, comparing the inhibitory potencies as has been done already for OCT2 (Hacker et al., 2015; Sandoval et al., 2018) and partially for OCT1 (Ahlin et al., 2011). This strategy has two advantages. First, using the victim drug of interest and not a model substrate enables more precisely predicting DDIs in humans. Second, and more interesting, it enables analyzing the interactions in the context of the specific substrate used. This may help identifying clusters of ligands (substrates and inhibitors) that potentially share binding positions in OCT1. In the long term, this may help to stratify ligands into groups according to similar binding properties and to generate subgroup-specific pharmacophores.

Secondly, comparing the interaction with OCT1, ligands with closely related structures may help to identify moieties that are important for the interaction. Systematic comparison of the OCT1 inhibitory potency of structurally similar morphinan opioids revealed that only minor structural changes, involving the ether linkage between C4-C5 of the morphinan ring, strongly increased the inhibitory potency for OCT1 (Meyer et al., 2019). Such systematic “ligand structure walking” may prove to be very useful to better understand the role of the ligand structure in the OCT1 transport mechanism.

All examples of experiments listed above are possible today due to technical advancement of the analytical methods. In contrast to the first decades of studying OCT1 where scientists were limited by the availability of radioactive OCT1 substrates, today we can use techniques such as LC-MS/MS to quantify practically any substrate of interest. The sensitivity is still not as high as in radioactive detection, but the quantification of the intracellularly accumulated substrate is highly specific. This enabled first high-throughput screens for OCT1 substrates (Hendrickx et al., 2013) and also detailed analyses of substrates with only slightly different chemical structures (Meyer et al., 2019) up to stereoselective effects of the uptake (Jensen et al., 2020).

Approaching polyspecificity from the transporter side, the species-specific differences in OCT1 transport can be used as a tool to identify domains or even single amino acids responsible for the substrate-specific effects on transport. In a proof-of-principle study we used human-mouse chimeric constructs to localize the cause for the higher affinity of mouse OCT1 for thiamine and metformin to transmembrane helices 2 and 3 (Meyer et al., 2020). For metformin, we were even able to fine-map the causal difference to the difference between Leu155 in human and Val156 in mouse OCT1. This strategy is extendable to all substrates showing species-specific differences in uptake. Furthermore, similar to the ortholog comparison, also paralogs with different substrate preferences may be compared, as has successfully been done for rat Oct1 and Oct2 (Gorboulev et al., 2005).

**FIGURE 1:**
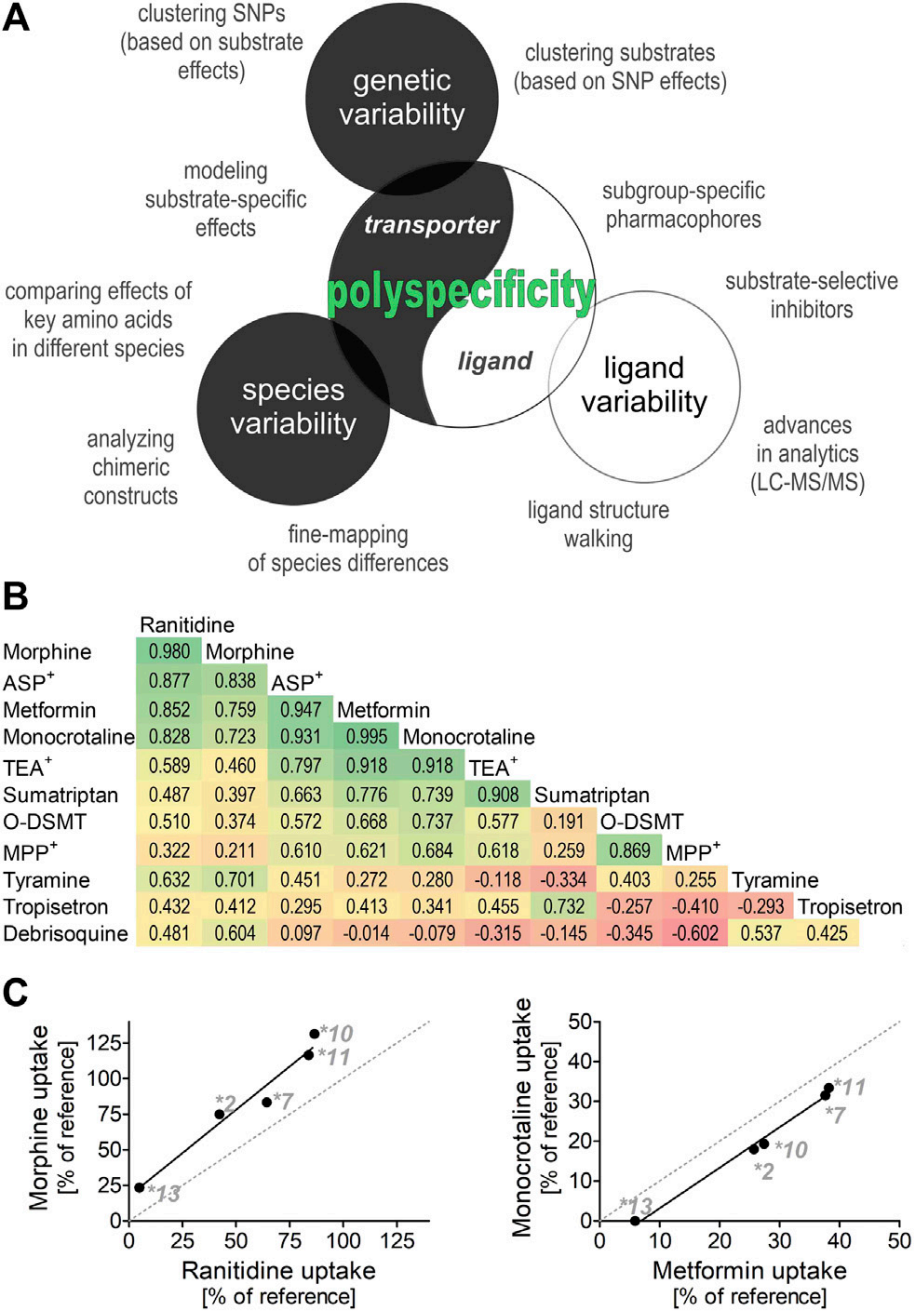
Polyspecificity as a friend **(A)** Illustrates different strategies for using polyspecificity as a tool to study the mechanisms of OCT1 transport. Given are ligand-based and transporter-based approaches, including the use of species and genetic differences. **(B)** and **(C)** Summarize and analyze data of five previous studies (Tzvetkov et al., 2012; Tzvetkov et al., 2013; Seitz et al., 2015; Matthaei et al., 2016; Meyer et al., 2017) as illustration of the use of SNP effects to cluster OCT1 substrates into different subgroups. Shown are the effects of OCT1 alleles *2, *7, *10,*11, and *13, which are known to have strongly substrate-specific effects on transport (Seitz et al., 2015), on the OCT1-mediated uptake of 11 substrates. The pairwise correlation coefficient between the effects of different alleles are given **(B)** and the two strongest correlations are shown **(C)** O-DSMT, O-Desmethyltramadol.

Similarly, substrate-specific effects of some OCT1 genetic variants may help to reveal details in the mechanism of OCT1 transport. By comparing the effects of genetic variants with substrate-specific effects on different substrates, we can identify substrates that are similarly affected and thus may share similar binding sites in the transporter. To illustrate this, we used previously published data about the effects of the substrate-specific OCT1 genetic variants OCT1*2, *7, *10, *11, and *13 from our group (Figures 1B,C (Tzvetkov et al., 2012; Tzvetkov et al., 2013; Seitz et al., 2015; Matthaei et al., 2016; Meyer et al., 2017)). The effects on the uptake of metformin and monocrotaline, but also of morphine and ranitidine correlated very well (r of 0.995 and 0.98, respectively; Figure 1), suggesting at least two groups of structurally divergent substrates that may share similar binding sites in OCT1. This strategy could be used to cluster ligands into subgroups based on the impact of the substrate-specific OCT1 genetic variants. Such subgroups could be used to develop subgroup-specific pharmacophores (similar to those suggested above for analyses of substrate-specific DDIs) and to identify subgroup-specific model substrates that will facilitate the handling of OCT1 pharmacogenetics in a clinical setting.

## Summary

The polyspecificity of OCT1 sets many hurdles for understanding the transport mechanisms of OCT1 and for the translation of our knowledge about OCT1 into clinical practice. However, polyspecificity may be used also as a tool, especially to reveal the mechanisms of OCT1 transport, which is an essential step for deepening our understanding of the physiological functions and potential pharmacological implications of this transporter.
